# Identification of KIF3A as a Novel Candidate Gene for Childhood Asthma Using RNA Expression and Population Allelic Frequencies Differences

**DOI:** 10.1371/journal.pone.0023714

**Published:** 2011-08-30

**Authors:** Melinda Butsch Kovacic, Jocelyn M. Biagini Myers, Ning Wang, Lisa J. Martin, Mark Lindsey, Mark B. Ericksen, Hua He, Tia L. Patterson, Tesfaye M. Baye, Dara Torgerson, Lindsey A. Roth, Jayanta Gupta, Umasundari Sivaprasad, Aaron M. Gibson, Anna M. Tsoras, Donglei Hu, Celeste Eng, Rocío Chapela, José R. Rodríguez-Santana, William Rodríguez-Cintrón, Pedro C. Avila, Kenneth Beckman, Max A. Seibold, Chris Gignoux, Salma M. Musaad, Weiguo Chen, Esteban González Burchard, Gurjit K. Khurana Hershey

**Affiliations:** 1 Division of Asthma Research, Cincinnati Children's Hospital Medical Center, Cincinnati, Ohio, United States of America; 2 Division of Biostatistics and Epidemiology, Cincinnati Children's Hospital Medical Center, Cincinnati, Ohio, United States of America; 3 The Lung Biology Center, San Francisco General Hospital, San Francisco, California, United States of America; 4 The Institute for Human Genetics, University of California San Francisco, San Francisco, California, United States of America; 5 Instituto Nacional de Enfermedades Respiratorias, Mexico City, Mexico; 6 Centro de Neumología Pediátrica, CSP, San Juan, Puerto Rico, United States of America; 7 San Juan Veterans Affairs Medical Center, University of Puerto Rico School of Medicine, San Juan, Puerto Rico, United States of America; 8 Division of Allergy-Immunology, Northwestern University, Chicago, Illinois, United States of America; 9 Biomedical Genomics Center, University of Minnesota, Minneapolis, Minnesota, United States of America; 10 Department of Medicine, National Jewish Health, Denver, Colorado, United States of America; 11 The Department of Biopharmaceutical Sciences, University of California San Francisco, San Francisco, California, United States of America; St. Petersburg Pasteur Institute, Russian Federation

## Abstract

**Background:**

Asthma is a chronic inflammatory disease with a strong genetic predisposition. A major challenge for candidate gene association studies in asthma is the selection of biologically relevant genes.

**Methodology/Principal Findings:**

Using epithelial RNA expression arrays, HapMap allele frequency variation, and the literature, we identified six possible candidate susceptibility genes for childhood asthma including *ADCY2*, *DNAH5*, *KIF3A*, *PDE4B*, *PLAU*, *SPRR2B*. To evaluate these genes, we compared the genotypes of 194 predominantly tagging SNPs in 790 asthmatic, allergic and non-allergic children. We found that SNPs in all six genes were nominally associated with asthma (p<0.05) in our discovery cohort and in three independent cohorts at either the SNP or gene level (p<0.05). Further, we determined that our selection approach was superior to random selection of genes either differentially expressed in asthmatics compared to controls (p = 0.0049) or selected based on the literature alone (p = 0.0049), substantiating the validity of our gene selection approach. Importantly, we observed that 7 of 9 SNPs in the *KIF3A* gene more than doubled the odds of asthma (OR = 2.3, p<0.0001) and increased the odds of allergic disease (OR = 1.8, p<0.008). Our data indicate that *KIF3A rs7737031* (T-allele) has an asthma population attributable risk of 18.5%. The association between *KIF3A rs7737031* and asthma was validated in 3 independent populations, further substantiating the validity of our gene selection approach.

**Conclusions/Significance:**

Our study demonstrates that *KIF3A*, a member of the kinesin superfamily of microtubule associated motors that are important in the transport of protein complexes within cilia, is a novel candidate gene for childhood asthma. Polymorphisms in *KIF3A* may in part be responsible for poor mucus and/or allergen clearance from the airways. Furthermore, our study provides a promising framework for the identification and evaluation of novel candidate susceptibility genes.

## Introduction

The amount of genetic information available with high throughput screens has increased exponentially in the last decade, with the potential for information on three billion base pairs being available through genome wide sequencing. While the genome wide approach has been successfully used to identify numerous genetic variants associated with complex human diseases [1], most variants identified so far confer relatively small increments in risk, and explain only a small proportion of disease heritability. This has led to considerable speculation regarding the sources of the “missing heritability” [2]. Common diseases such as asthma are heterogeneous and may actually be a compilation of disorders with numerous subphenotypes. Current studies are not designed to examine specific subphenotypes of disease due to the large sample sizes required for genome wide approaches. Genome wide approaches require thousands of cases and controls to have sufficient power to properly evaluate such associations. As with many complex diseases such as asthma, analysis of large sample sizes made up of heterogeneous phenotypes may make it more difficult to identify true associations. By focusing on regions with *a priori* evidence of gene involvement, the candidate gene approach has the advantage of requiring smaller sample sizes as fewer statistical tests are performed. One of the major challenges of the candidate gene approach for genetic studies is selection of appropriate genes for evaluation. Methods that have been utilized thus far include selection of genes based on published biologic function, findings from mouse models of asthma, and chromosomal location in ‘hot-spots’ that have been linked to disease phenotypes in genome wide association studies (GWAS) and linkage studies. While over 600 studies have used these strategies to identify more than 120 different genes to be associated with asthma or its related phenotypes, only a limited number of genes have been replicated leaving much variation yet unexplained [3].

Another promising strategy to narrow the numerous potential disease-associated genes in a less biased way involves examining differences in allele frequencies between populations in conjunction with differences in gene expression within relevant cell types or tissues [4]. Inclusion of an analysis of allele frequency differences between populations may be a beneficial strategy for any disease that shows significant differences in prevalence between groups. A recent study by Frank et. al [5] integrated global gene expression arrays, DNA sequence variation arrays, and public databases to identify new previously untested candidate genes for further testing. This more targeted approach allows researchers to reduce the overall number of genes to be tested and hence increases statistical power to detect an association by lowering the multiple testing burden.

The overall objective of our study was to develop an innovative approach for identifying candidate genes for genetic association studies with complex diseases including asthma. We combined the unbiased characteristics typically obtained using GWAS or expression arrays with the more focused quality of traditional literature-based candidate gene approaches. The basis of our resulting approach takes advantage of our previously published evaluation of nasal epithelial cell-derived RNA from asthmatic and non-allergic children [6], population differences in asthma prevalence, tagging SNPs in the HapMap database, and the published literature. Importantly, nasal epithelial cell samples were used as our source tissue, because of their ability to interface with and function as a physical barrier to the environment, their importance in initiating the immune response to environmental triggers and their role in modulating allergic inflammation [7], [8]. Further, compared to collection of bronchial lavage fluid or bronchial biopsies, nasal epithelial cell collection is less invasive and has been shown to be a good surrogate for the lower airway epithelium [9], [10].

Using our approach, we identified five novel genes that had not been previously implicated in asthma and a sixth gene (*PDE4B*) that was recently linked to asthma (Table 1) [11]. Upon analysis, we determined that all six genes were nominally associated with pediatric asthma in our discovery population (p<0.05). SNPs in a single gene, *KIF3A*, were significant even after considering multiple comparisons (p<0.0001). Our results substantiate the validity of our candidate gene selection approach.

**Table 1:** Selected genes and functions.

#SNPs^a^	Gene	Full Gene Name	Chr.	Array Findings^b^	Reported Processes and Function^c^	Reported Associated Diseases
9	*KIF3A*	kinesin family member 3A	5q31	Down	protein binding, ATP binding, microtubule motor activity, nucleotide binding	
53	*DNAH5*	dynein, axonemal, heavy polypeptide 5	5p15	Down	microtubule motor activity, ATP binding, ATPase activity, nucleotide binding	primary ciliary dyskinesia, ciliary motility disorders
68	*ADCY2*	adenylate cyclase 2	5p15	Down	adenylate cyclase activity, magnesium ion binding, phosphorus-oxygen lyase activity	
7	*PLAU*	plasminogen activator, urokinase	10q24	Up	kinase activity, peptidase activity, plasminogen activator activity, serine-type endopeptidase activity	acantholysis, alzheimers rheumatoid arthritis, cancer, endometriosis
53	*PDE4B*	phosphodiesterase 4B	1p31	Up	3′,5′-cyclic-AMP phosphodiesterase activity, 3′,5′-cyclic-nucleotide phosphodiesterase activity, catalytic activity, hydrolase activity	chronic kidney failure, schizophrenia
4	*SPRR2B*	small proline-rich protein 2B	1q21	Up	structural molecule activity, keratinization	

a Indicates the total number of genotyped SNPs.

b Indicates the direction of gene expression of uncontrolled asthmatics versus non-allergic controls.

c Obtained from Gene Ontology website (www.geneontology.org).

## Methods

### Ethics

The study protocol was approved by the Cincinnati Children's Hospital Medical Center Institutional Review Board. Parents gave written informed consent for the children's participation, and children gave their assent.

### Study Populations

The discovery population consisted of a subset of 4 to 17 year old Caucasian participants enrolled in either the Greater Cincinnati Pediatric Clinic Repository (GCPCR) or the Genomic Control Cohort (GCC), a cohort supported by the Cincinnati Children's Hospital Medical Center (CCHMC). The GCPCR includes over 6,200 patients with various diagnoses visiting CCHMC outpatient specialty clinics, the Emergency Department or recruited from the community. Participants completed questionnaires, and provided buccal or saliva samples for genetic analyses. The GCC includes over 1,080 children recruited to be representative of the Greater Cincinnati area. All GCC participants completed a questionnaire that included asthma, allergy and skin questions similar to those included in GCPCR and provided a blood sample for genetic analysis. Non-asthmatic/non-allergic control children were not proactively recruited into the GCPCR for this study due to the availability of appropriate GCC controls. GCC asthmatics were not included in the analyses of the discovery cohort, because their asthma diagnoses were based on parent report alone. Inclusion criteria and case-control definitions are described in Table 2.

**Table 2:** Inclusion criteria of discovery and replication study populations.

Population	Race or Ethnicity	Population Source(s)	# asthmatics	# allergic	# controls	Determination of race or ethnicity	Asthma inclusion criteria	Allergic inclusion criteria	Control inclusion criteria
Discovery	Caucasian	Greater Cincinnati Pediatric Clinic Repository (GCPCR), CCHMC Genomic Control Cohort (GCC)	312	220	246	Race was self-reported and ascertained by questionnaire; both the participant and his/her parents must have been identified themselves as Caucasian/White; all subjects were non-Hispanic	Children 4–17 years old with confirmed physician diagnoses of asthma based on clinical examination, available pulmonary function test results and respiratory symptom scores at a CCHMC-based specialty clinic	Non-asthmatic children ages 4–17 years old with physician diagnosed allergic rhinitis or atopic dermatitis based on radioallergosorbent testing or skin prick allergy testing; or children with a personal history of either environmental allergies, hay fever or eczema	Non-allergic Caucasian controls were those children ages 4–17 years old that did not meet the criteria to be either an asthmatic or allergic case, had no personal history of food allergies and no family history (parents and siblings) of asthma
Replication	African-American	GCPCR, GCC	182	129	39	Race was self-reported and ascertained by vquestionnaire; both the participant and his/her parents must have been identified themselves as African American/Black; all subjects were non-Hispanic	See Discovery Caucasian population above	See Discovery Caucasian population above	See Discovery Caucasian population above
	Caucasian	GCC, Cincinnati Control Cohort (CCC)	74	NA^a^	211	Parents of asthmatics reported their children to be Caucasian by questionnaire; adult controls similarly reported their race to be Caucasian by questionnaire	Caucasian children ages 4 to 18 years with parent-reported asthma from a population-based representative sample from Greater Cincinnati	NA	Caucasian adults ages 24 to 90 years with no personal or family history of asthma (as determined by self-report) from a population-based representative sample from Greater Cincinnati
	Puerto Rican	Genetics of Asthma in Latino Americans (GALA) Study	398	NA	712	Ethnicity was self-reported and ascertained by questions; both biological parents and all biological grandparents of asthmatics had to be identified as being of Puerto Rican ethnicity	Index children at least 8 years of age with physician diagnosed asthma (confirmed by hospital-based medical chart review) and two or more parent-reported asthma symptoms (among wheezing, coughing, and shortness of breath) in the last 2 years were enrolled over a 4-year period in the San Francisco Bay Area, California, New York City, New York , Puerto Rico, and Mexico City, Mexico	NA	One or both biological parents of index asthmatic children were enrolled; allergic status was not a criteria for inclusion or exclusion
	Mexican	GALA	300	NA	585	Ethnicity was self-reported and ascertained by questionnaire; both biological parents of asthmatic children and all biological grandparents had to be identified as being of Mexican ethnicity	See Puerto Rican population above	NA	See Puerto Rican population above

a Not applicable.

In addition to our discovery population, four additional populations were used to replicate our findings (see Table 2). Cincinnati-based African American children selected from the GCPCR and GCC were identified as described in our discovery Caucasian population. Cases for a second Greater Cincinnati Caucasian population consisted of children with parent-reported asthma from the GCC and compared to the Cincinnati Control Cohort (CCC), a population based cohort of Caucasian adults with no personal or family history of asthma (by self-report) representative of Greater Cincinnati.with no personal or family history of asthma (by self-report) from a population-based representative adults sample from Greater Cincinnati. These controls were chosen because we could conclusively indicate their absence of pediatric and adult asthma, unlike similar aged controls that may develop asthma with age. Genotyping data from Affymetrix 6.0 SNP chip was also available for the GCC and CCC. The third and fourth populations consisted of Banked DNA was utilized for genotyping of Puerto Ricans and Mexicans parent- child trios with banked DNA for genotyping participating in the Genetics of Asthma in Latino Americans (GALA) Study [12], a multicenter international collaborative effort designed to identify clinical and genetic risk factors associated with asthma.

### DNA Isolation and Genotyping

Genomic DNA was isolated from buccal swabs with either the Zymo Research Genomic DNA II Kit (Zymo Research Corp., Orange, CA) or the Purgene DNA Purification System (Gentra Systems Minneapolis, MN), and from Oragene saliva samples per the kit's instructions. Alternatively, genomic DNA was extracted from blood samples using Manual PerfectPure DNA Blood Kit (Invitrogen, Carlsbad, CA). Genotyping from discovery cases and controls and African American samples was performed using a custom Illumina Golden Gate assay according to manufacturer's protocol (http://www.illumina.com; San Diego, CA). Genotypes were assigned using BeadStudio's genotyping module (BeadStudio v3.2, San Diego, CA). For GALA, genotyping of *KIF3A rs7737031* was accomplished using the Roche LightTyper 480 (Roche Diagnostics, Indianapolis, IN) and the *KIF3A rs7737031* using TaqMan SNP Genoptyping Assay (assay ID C_25973778_10; Applied Biosystems, Foster City, CA).

### Statistical Analysis

#### Genetic Association

SNPs failing Hardy Weinberg Equilibrium in the non-allergic control group (p<0.0001), having minor allele frequencies below 10%; or missing call rates greater than 10% were excluded. In addition, individuals with more than 20% of their total SNPs missing were excluded. Principal component analyses were performed using the 30 included ancestry-informative markers (AIMs) and the computer program EIGENSTRAT [13], [14] to account for potential population stratification. When examining the 194 SNPs in the six genes, the genomic inflation factor was 1.0, suggesting minimal impact of population stratification for these 30 AIMs. Therefore, no population stratification adjustment was required for analyses of asthmatics versus non-allergic controls in the discovery or African-American populations likely due to the fact that selection of the study population was from a single geographic region. However, the first principal component score was included as a covariate in comparisons of allergic versus non-allergic children in the discovery population as the genomic inflation factor was greater than 1.0 (λ = 1.33 before adjustment and λ = 1.02 after adjustment). Using PLINK [15], associations with asthma were tested adjusting for age and gender using the additive logistic regression model stratified by race. To address multiple testing, we first determined the average pairwise LD (a measured by r^2^) for all SNP combinations (160 SNPs, correlation = 0.13) and using this correlation, calculated the Bonferroni correction using the freely available Simple Interactive Statistical Analyses Software (http://www.quantitativeskills.com/sisa/). Associations were therefore considered significant at or below the 0.0006 level. For our second Caucasian population, six of the seven SNPs were imputed from the Affymetrix® 6.0 SNP data using MACH and HapMap CEU (release 22) as the reference [16], [17]. Imputed *KIF3A* SNPs were tested for association with asthma status again using additive logistic regression models in PLINK. The family-based association test (FBAT) [18] was used to assess associations between *KIF3A* rs7737031 and asthma in the GALA trios. Mexican and Puerto Rican samples were analyzed independently. HWE was tested in parents only. The population attributable risk for *KIF3A* rs7737031 was estimated using the R software package pARccs (v0.2–2; www.r-project.org) [19].

#### SNP Imputation

Estimation of SNPs not genotyped in the study was performed to increase statistical power as well as to detect novel associations^26^. Based on HapMap CEU results (release 22), imputation was performed after filtering out SNPs with genotyping call rates <10%, minor allele frequencies <10%, and HWE p-value<0.0001 using MACH 1.0.16 (http://www.sph.umich.edu/csg/MaCH), which uses a hidden Markov model to estimate an underlying set of unphased genotypes for each subject. We only considered SNPs that could be imputed with relatively high quality (RSQ>0.4). For the replication populations and validation of our approach, the available Affymetrix 6.0 SNP chip genotype data (http://www.ncbi.nlm.nih.gov/gap) from the populations described above were used. Imputed SNPs were tested for association with asthma status using PLINK software as described above.

#### Validation

To test whether F_ST_ is a proxy for minor allele frequency (MAF), we estimated Spearman correlation coefficients, as the MAF and F_ST_ were not normally distributed. For the comparison of SNPs with high and low F_ST_ values, pair-wise F_ST_ was determined using Python (http://www.python.org) scripts and HapMap data [20]. We selected 24 genes containing SNPs with the largest F_ST_ values (range 0.25–0.73) and 24 genes containing SNPs with the lowest F_ST_ values (range 0.00–0.10). We performed a case control analysis between self-reported child asthmatics from the GCC (n = 74) and controls with no personal or family history of asthma from the GCC (n = 226) using all Affymetrix 6.0 SNPs within 1000 kb of these genes. We then tested whether SNPs with high F_ST_ values (F_ST_≥0.1) are more likely to show association with asthma (p≤0.05) than SNPs with low F_ST_ values (F_ST_ = 0). To test whether our gene selection strategy was superior to random selection, we again evaluated the available Affymetrix data from the 74 GCC asthmatics and 238 CCC controls. From this analysis, four of our six genes exhibited significant evidence of association (p≤0.05). Results from this analysis were then utilized to select six genes from the 161 differentially regulated genes using a random number generator. We noted how many of the six genes had at least one SNP with nominal association (p≤0.05). We repeated this analysis 10,000 times. To determine the empirical level of significance, we determined how many times the random selection obtained evidence in at least four genes added one and divided by the number of replicates (10,000).

### Gene Expression Studies

Balb/c mice (Jackson Labs; Bar Harbor, ME) were sensitized twice intraperitoneally with 10 µg house dust mite (HDM, *Dermatophagoides pteronyssinus*; Greer Laboratories, Lenoir, NC) in 100 µl phosphate buffered saline (PBS) or 100 µl of PBS alone, and then challenged intratracheally with 100 µg HDM in 50 µl PBS or 50 µl PBS alone, euthanized 24 hours later, lung RNA extracted in TRIzol (Invitrogen, Carlsbad, CA) and cDNA prepared (Superscript First Strand cDNA synthesis kit; Invitrogen, Carlsbad, CA). Quantitative PCR on mouse and human cDNA samples collected previously [6] were performed using the Roche Light Cycler 480 SYBR Green 1 Master kit (Mannheim, Germany). The annealing temperature for primers sets was 60°C and the extension time was 5 seconds. After amplification, values from *KIF3A* were normalized to respective housekeeping genes values (glyceraldehyde 3-phosphate dehydrogenase (*GAPDH*) or *actin*). Primers used to amplify both human and mouse *KIF3A* include: Forward- 5′-GGAGGAGACGAGCTGAG-3′; Reverse- 5′-CTCTGACTTTGCAGCCA-3′. Primers used to amplify human GAPDH: Forward- 5′-AAATCCCATCACCATCTTCC-3′; Reverse- 5′-TCACACCCATGACGAACA-3′. Primers used to amplify mouse actin: Forward - 5′-GGCAATGCGGCTGCAA-3′; Reverse- 5′-GGGTACCCACGCGAATCAC-3′. For the human epithelial gene expression studies, statistical analysis was performed using PRISM software (GraphPad Software Inc., La Jolla, CA) using one-way ANOVA followed by a Tukey-Kramer post-hoc test (for statistical significance between groups). For comparisons showing significant differences, precise p-values were calculated using a two-tailed t-test comparing the two groups under consideration. In the mouse studies, statistical significance was determined using a two-tailed t-test in PRISM.

## Results

### Gene Selection Approach

**Figure 1:**
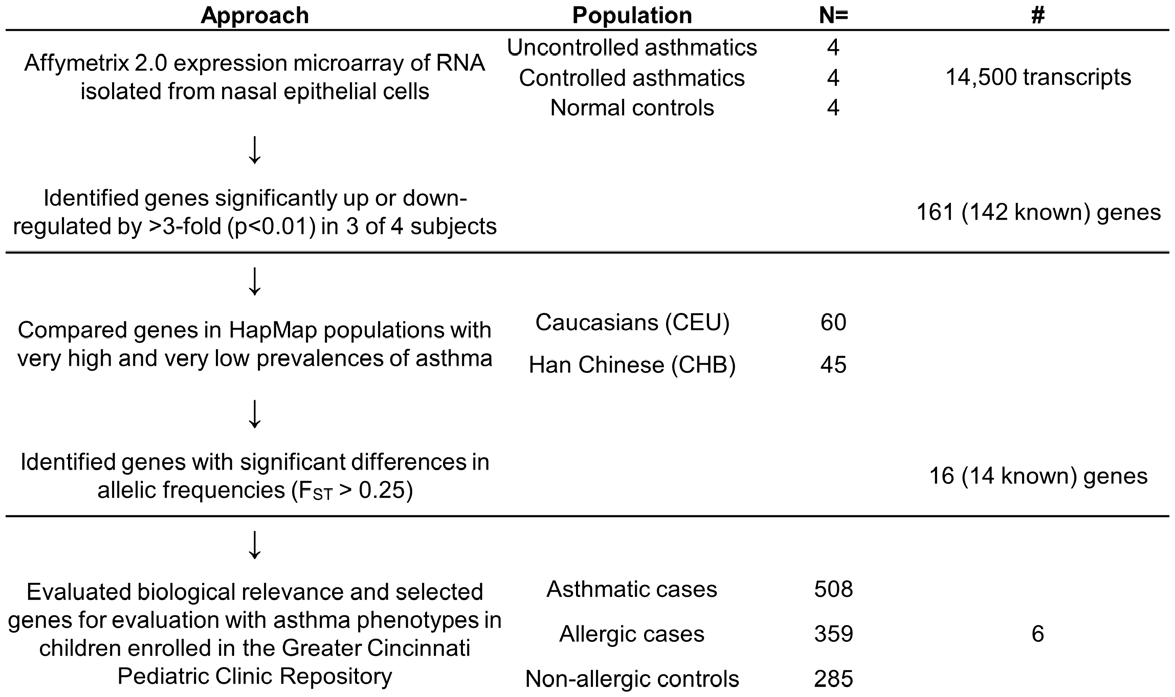
Novel unbiased approach to identify candidate asthma susceptibility genes. The approach consisted of three stages with an evaluation of RNA expression of 14,500 genes in nasal epithelial samples in stage 1. Next, 142 known genes with >3-fold difference between uncontrolled asthmatics and non-allergic controls (P<0.05) were taken forward to stage 2. In stage 2, using HapMap data, the allelic frequencies of Caucasians (Utah residents of European ancestry; CEU) and the Han Chinese (Beijing, China; CHB) were compared and 14 known genes with a fixation index (F_ST_)>0.25 were identified. Six of these genes, which mapped to chromosomal regions that had been linked to asthma previously, were included in the next phase. In stage 3, tagging SNPs including all CEU and YRI (Yoruban residents of Ibadan, Nigeria) SNPs with minor allele frequency less than 0.05 in the 6 genes were genotyped in children with asthma, allergic rhinitis or atopic dermatitis without asthma, and in non-allergic control children using a custom Illumina Golden Gate SNP Chip. Seven SNPs in a single gene, *KIF3A*, were significantly associated with asthma after adjusting for multiple comparisons (p-value<0.0006).

We previously published a RNA expression study in nasal epithelial samples from uncontrolled and controlled pediatric asthmatics compared to non-allergic controls [6]. The results of our study revealed that compared to the non-allergic control group (n = 4), the mean gene expression levels of 161 genes (142 known genes) were consistently up or down regulated in the group of uncontrolled asthmatics (n = 4) (one-way ANOVA ; p<0.01) (Fig. 1). To further reduce the pool of 161 candidate genes identified, we compared allele frequencies of SNPs within these genes in two distinct populations shown to have large differences in population prevalence of asthma. We hypothesized that specific alleles in genes with large inter-population frequency differences might be partly responsible for observed variations in asthma susceptibility. In fact, a recently published genome-wide estimation of the fixation index (F_ST_), a measure of population differentiation devised by Wright [21], [22], on approximately 4 million SNPs from the HapMap project, found that genes associated with complex diseases showed a significantly higher mean value of F_ST_ suggesting that population genetic differentiation, particularly in genes associated with complex diseases may explain discrepancies in disease prevalence between different populations [23].

For our analysis, we used Wright's F_ST_ to quantify genetic differentiation between the Phase I HapMap CEU (Utah residents with Northern and Western European ancestry, n = 60) and the CHB (Han Chinese in Beijing, China, n = 45). We selected these populations based on the largest reported disparity in asthma prevalence in the ISAAC report [24] available at the time among the four Phase I HapMap populations (≥10% asthma prevalence for Caucasians vs. <5% for Chinese, respectively). F_ST_ was calculated for individual SNPs (F_ST_ = (*p_1_*−*p_2_*)^2^/(4*p*(1−*p*)), where *p_1_* is the allele frequency in the Caucasian population, *p_2_* is the frequency of the same allele in the Han Chinese population and *p* is the average allele frequency of each allele across each population [25], [26]. As F_ST_ has a theoretical minimum of 0 indicating no genetic differences, and a theoretical maximum of 1 indicating fixation for alternative alleles, we hypothesized that genes with larger F_ST_ values between populations that differ in disease prevalence are more likely to be associated with disease. Using this approach, we identified 16/161 genes (or 14/142 known genes) showing differential expression between cases and controls to have relatively large differences in allele frequencies in least one SNP (F_ST_≥0.25; Fig. 1).

Next, these 16 genes were subjected to an extensive literature review using publicly available databases such as PubMed. Our investigation revealed that six of these genes (*PDE4B*, *SPRR2B*, *ADCY2*, *KIF3A*, *DNAH5*, and *PLAU*; see Table 1) were located in chromosomal regions that had been previously linked to asthma or other allergic disease phenotypes and had been shown to be regulated during allergic inflammation [1] (Table 1). Furthermore, we identified five of the same six genes (*ADCY2*, *DNAH5*, *KIF3A*, *PDE4B*, *SPRR2B*) when substituting F_ST_ values calculated using the HapMap CEU and YRI (Yorubans from Ibadan, Nigeria) populations for the CEU and CHB populations.

### Tagging SNP Selection

Consequently and independent of the SNPs evaluated in our analysis of F_ST_, a total of 172 tagging SNPs were selected for inclusion on a custom Illumina Golden Gate platform for the six genes of interest using Haploview and Tagger (http://www.broad.mit.edu/mpg/haploview). All tagging SNPs included were required to have minor allele frequencies greater than 0.05 and patterns of linkage disequilibrium (LD; r^2^>0.8) in the public HapMap Phase I CEU and YRI populations (http://hapmap.ncbi.nlm.nih.gov) [27], [28], [29].The rationale for using tagging SNPs is that genetic variants that are near each other and in LD tend to be inherited together as a result of shared ancestry. The strong correlations between markers within haplotype blocks help to enable accurate representation of a gene region by a small number of tagging SNPs and further ensures the efficient capture all the common genetic variation in the genes selected. We selected SNPs from the CEU and YRI populations because our discovery population consisted of children of Caucasian ancestry, and the availability of African American children for a replication cohort. We also included an additional 18 non-synonymous SNPs, four promoter SNPs, and 30 unlinked ancestry-informative markers (AIMs) [30] to estimate global population structure. AIMs were selected based on the criterion previously described by *Rosenberg et al., 2003* [30]. Twenty of these AIMs were included to specifically distinguish between Northern and Southern Europeans [31] and the remaining ten AIMs were chosen to specifically distinguish between Europeans and people of African descent.

### Asthma and Allergic Disease Genetic Associations

Genotyping was performed first on 790 Caucasian children from the Cincinnati Metropolitan area. Our SNP call rate was uniformly >95%. SNPs with call rates <10% (N = 9) or minor allele frequencies <10% (N = 25) were removed from the analysis. None of the SNPs failed Hardy-Weinberg Equilibrium in the non-allergic control population (p<0.001). When the more conservative cut off of 0.01 was used, only three additional SNPs would have been excluded. Individuals with more than 20% of their total SNPs missing were also excluded from the analysis (N = 12; Table 3). The mean age of the remaining children was slightly, but significantly less for asthmatic (p = 3.9×10^−9^) and allergic (p = 1.5×10^−6^) children compared to the non-allergic control group (Table 3).

**Table 3:** Characteristics of the discovery Caucasian population.

	Asthmatic Children	Allergic Children	Non-allergic Controls
Total children, N	317	227	246
Children after exclusions, N^a^	312	220	246
Mean age (years)± SD	10.04±3.44^b^	10.21±3.54^b^	11.79±3.40
Male gender, N (%)	170 (54.5)	126 (57.3%)	121(49.2)

a Indicates the number children after children with missing call rates above 20% were removed.

b Indicates significant differences (p<0.05) with non-allergic control children.

**Figure 2:**
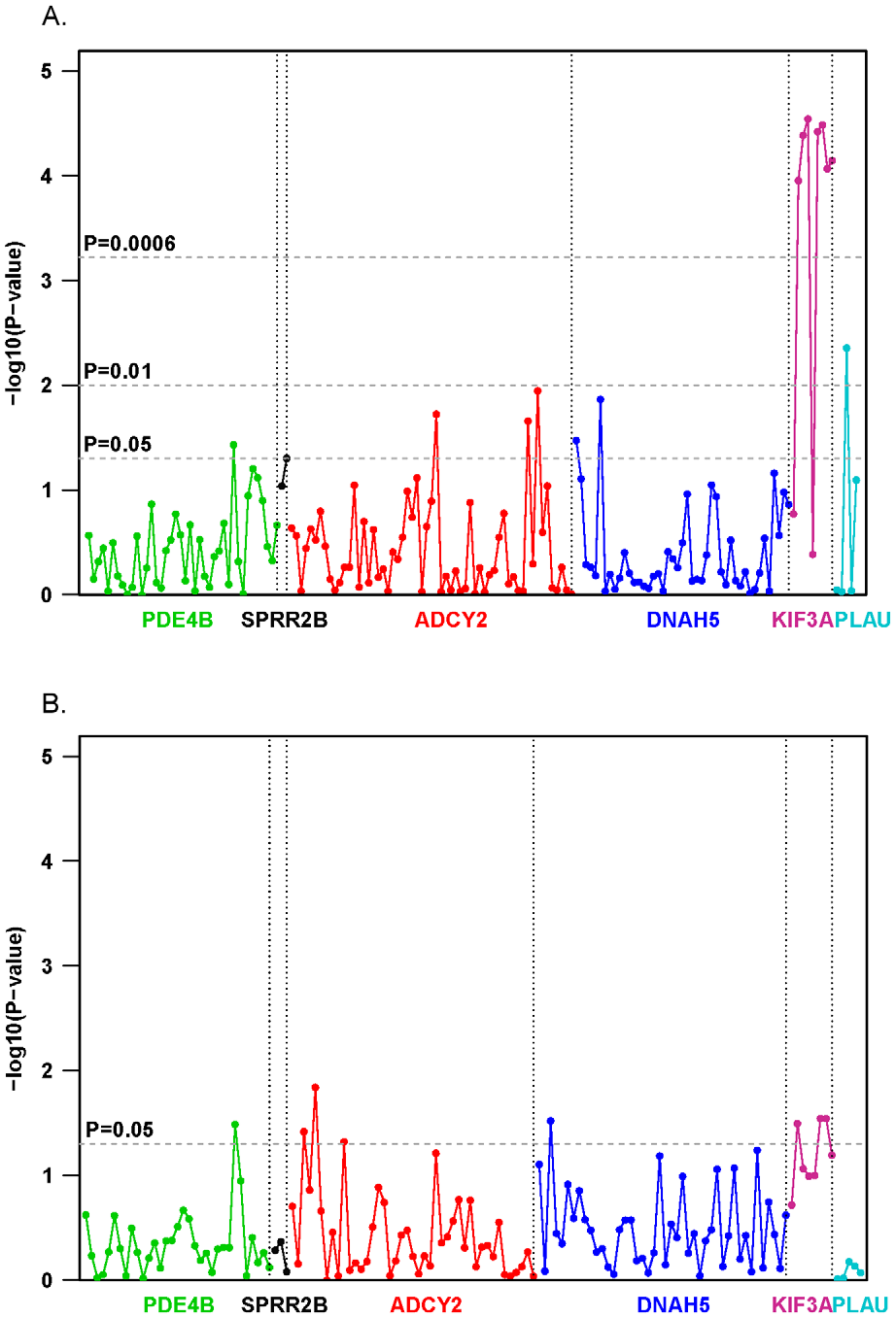
Genetic associations with childhood asthma. **A**. We evaluated associations in our discovery Caucasian population between asthma and 160 directly genotyped SNPs within the six epithelial genes using the additive model after adjusting for age and gender. The upper dashed line corresponds to a p-value of 0.0006, the Bonferroni adjustment after considering LD correlation between SNPs. SNPs significant at this level (all in *KIF3A*) include rs12186803 (p = 0.00011), rs3798130 (p = 0.00004), rs2299011 (p = 0.00003), rs12514685 (p = 0.00004), rs7737031 (p = 0.00003), rs1080001 (p = 0.00009), and rs9784675 (p = 0.00007). The lower dashed line corresponds to a p-value of 0.05. SNPs significant at this level include rs11747117 (p = 0.0188), rs7714830 (p = 0.0219), and rs13174121 (p = 0.0113) in *ADCY2*, rs2896111 (p = 0.0335) and rs17263496 (p = 0.0136) in *DNAH5*, rs12060491 (p = 0.0369) in *PDE4B*, rs6693927 (p = 0.0496) in *SPRR2B* and rs2227562 (p = 0.0044) in *PLAU*. **B**. Associations between asthma and 160 directly genotyped SNPs within the six epithelial genes were evaluated among African American children from Cincinnati using an additive model after adjusting for age and gender. The dashed line corresponds to a p-value of 0.05. SNPs significant at this level include rs11742602 (p = 0.038), rs2017214 (p = 0.014) and rs1032719 (p = 0.048) in *ADCY2*, rs30168 (p = 0.030) in *DNAH5*, rs11208834 (p = 0.032) in *PDE4B*, rs12186803 (p = 0.032), rs1080001 (p = 0.029) and rs7737031 (p = 0.028) in *KIF3A*.

Using a case-control study design, associations with asthma were evaluated. Notably, seven of nine *KIF3A* SNPs were associated with asthma with more than a 2-fold increase in the odds ratio for asthma (p<10^−4^) even after applying an LD adjusted Bonferroni correction for 160 pairwise comparisons with 0.13 correlation (α = 6×10^−4^; Table 4). We retrospectively compared the F_ST_ estimates of differentiation between HapMap (Phase III, Build 26) CEU and CHB populations for each of the *KIF3A* SNPs genotyped (Table 4). Our analysis revealed that all seven of the asthma-associated *KIF3A* SNPs had F_ST_ values≥0.32, while the remaining 2 SNPs had much lower F_ST_ estimates, suggesting that F_ST_ may be useful in identifying those SNPs most likely to be associated with asthma. Interestingly, at least one genetic variant in each of the six identified epithelial genes was nominally associated with asthma at p-value<0.05 (Fig. 2A).

**Table 4:** *KIF3A* SNP associations with asthma.

Population =			Discovery Caucasian^a^		African-American^a^		Secondary Caucasian^b^		Puerto Rican^c^	Mexican^c^
# cases/# controls =			312/246		182/39		74/211		398	300
*KIF3A* SNP	Major/Minor Allele	F_ST_ e	OR	P-value	OR	P-value	OR	P-value	P-value	P-value
rs12186803	G/A	0.33	2.08	**0.00011**	1.79	**0.032**	3.21	**0.005**		
rs1080001	A/G	0.32	2.08	**0.00009**	1.83	**0.029**	2.76	**0.011**		
rs7737031	C/T	0.34	2.18	**0.00003**	1.83	**0.028**	2.76	**0.011**	**0.0458**	0.471
rs9784675	A/G^d^	0.33	2.09	**0.00007**	1.61	0.064	3.32	**0.002**		
rs3798130	G/A^d^	0.33	2.16	**0.00004**	1.56	0.086	3.21	**0.005**		
rs2299011	C/G^d^	0.32	2.19	**0.00003**	1.52	0.101	3.21	**0.005**		
rs12514685	C/T^d^	0.32	2.16	**0.00004**	1.52	0.100	2.76	**0.011**		
rs1468216	G/A	0.01	0.78	0.16870	0.68	0.191	1.08	0.810		
rs17691077	A/C	0.06	0.85	0.41000	-	-	-	-		

a The discovery Caucasian population and African American population consisted of asthmatic children from the GCPCR and non-asthmatic/non-allergic controls from the GCPCR and GCC. Associations between asthmatics and controls were tested using an additive model. Odds ratios (OR) were determined using logistic regression based on the minor allele after adjusting for age, gender and population stratification. Bolding indicates the SNP associations <0.05. 7 of 9 SNPs were significant even after considering multiple comparisons (p<0.0006).

b The Secondary Caucasian population consisted of asthmatic children from the GCC and non-asthmatic adult controls from the CCC. OR and p-values were determined using HapMap CEU results (release 22) and Affymetrix data from each cohort for imputation analysis.

c The Puerto Rican and Mexican populations consisted of mother, father, child trios enrolled in the GALA Study. The value provided is the frequency of childhood asthmatics within those trios.

d Major and minor alleles are reversed in African Americans;

e Value of fixation index (F_ST_) between CHB (China/Beijing) and CEU (Caucasian Europe, as represented by Utah) HapMap populations.

To further verify our findings and substantiate the approach, we evaluated associations with asthma in 362 African American children also from the Cincinnati Metropolitan area. We found that *ADCY2*, *PDE4B*, *DNA*H5 and *KIF3A* were again associated with asthma (p<0.05) at the gene level (Fig. 2B). Interestingly, seven *KIF3A* SNPs were also significantly associated with other allergic diseases such as allergic rhinitis and eczema (p-values≤0.008 and p-value≤0.023, respectively) in both our discovery Caucasian and African American populations (Table 4).

**Figure 3:**
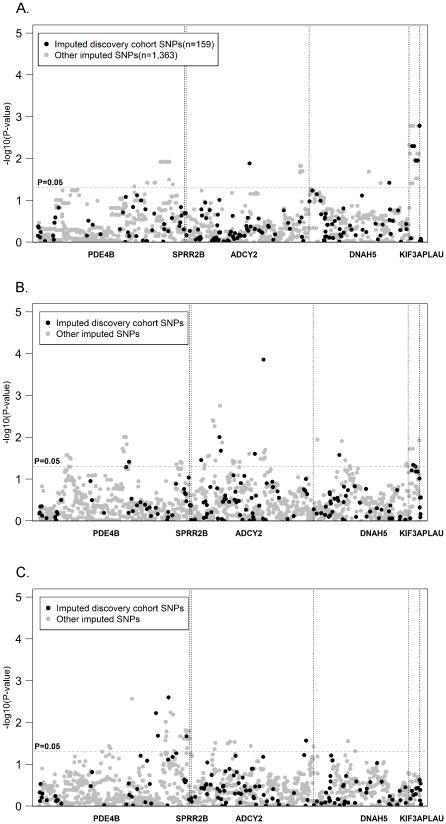
Associations with asthma in three additional independent populations. SNPs not genotyped in the study were imputed based on HapMap CEU results (release 22) with MACH software using the available from the Affymetrix 6.0 genotype data. Imputed SNPs were then tested for associations with asthma using PLINK. The dashed line corresponds to a p-value of 0.05. **A**. Associations in a second Cincinnati-based Caucasian population. **B**. Asthma associations in the GALA Puerto Rican trios. **C**. Associations in the GALA Mexican trios.

In both our discovery Caucasian and our African American populations, asthma was diagnosed according to ATS criteria [32] by hospital-based specialists at CCHMC. In order to validate the results we had obtained from these subsets, we utilized childhood and adult population-based cohorts for which Affymetrix 6.0 gene chip genome-wide genotyping data was available. We evaluated associations between our candidate genes and parent reported diagnosis of asthma in 74 Caucasian childhood asthmatics and 211 adults with no personal or family history of asthma living in Cincinnati. We performed imputation analysis to directly assess associations of tagging SNPs previously genotyped in our discovery Caucasian population as well as other SNPs not previously genotyped. Our analysis revealed all 7 previously associated *KIF3A* SNPs (as well as several other imputed SNPs) were significantly associated with asthma in this population (p-value≤0.01; Table 4). Furthermore, at least one previously associated SNP within *ADCY2 and DNAH5* was associated with asthma (Fig. 3A). Imputed SNPs within *PDE4B* not previously genotyped were also significantly associated with asthma.

We next examined associations between imputed SNPs and asthma in 398 Puerto Rican and 300 Mexican family trios enrolled in the Genetics of Asthma in Latino Americans (GALA) Study [12]. Puerto Ricans living in the US have higher asthma prevalence (26%), morbidity, and mortality rate than Caucasians living in the US, and Mexicans living in the US have a lower asthma prevalence (10%), morbidity, and mortality rate [33], [34], [35], [36], [37], [38]. Our analysis provided evidence for gene level replication of *ADCY2, DNAH5, KIF3A, and PDE4B* (Fig. 3B, C, respectively). As only *KIF3A* achieved significance after correction for multiple testing, this replication is essential to minimize false positive findings. To further support our findings, we directly genotyped *KIF3A* rs7737031, the SNP with the most significant association in our discovery population, and again observed a significant association with asthma in the Puerto Rican (p-value = 0.0458), but not the Mexican trios (p-value = 0.471) (Table 4).

### Validation of Candidate Gene Selection

To validate our approach, we tested whether identifying candidate genes based on higher values of F_ST_ is a proxy for identifying more common SNPs with more power to detect an association, or whether information is gained from measures of population differentiation. We found that F_ST_ values for the 161 differentially expressed genes in uncontrolled asthma were not significantly correlated with minor allele frequency (Spearman rho = 0.05, p-value = 0.52), suggesting that including measures of F_ST_ to identify candidate genes is not simply identifying SNPs with greater power to detect an association, but rather provides information beyond that of minor allele frequency.

Second, we compared the HapMap CEU and CHB populations and determined whether SNPs with high F_ST_ values were more likely to exhibit association with asthma than those SNPs with low F_ST_ values. We evaluated Affymetrix 6.0 data of 74 asthmatic and 238 non-allergic control children from Cincinnati. Of the 161 differentially regulated genes identified by our expression array, we determined that the 241 SNPs within the 24 genes with the highest F_ST_ values (F_ST_≥0.1; including SNPs within the final six genes selected - *KIF3A*, *ADCY2*, *DNAH5*, *PDE4B*, *PLAU*, *SPRR2B*) were significantly more likely (p = 0.0008, Fisher's Exact Test; 6% of SNPs reached p≤0.05) to be associated with asthma than the 190 SNPs in the 24 genes with the lowest F_ST_ values (F_ST_ = 0; 0.5% of SNPs reached p≤0.05).

Finally, again using the Affymetrix 6.0 data of the 74 Caucasian asthmatic children and 238 control children described above, we randomly selected six genes among those differentially expressed in asthmatics versus controls and asked how often we would observe at least four of the six genes having at least one SNP significantly associated with asthma (p-value = 0.05). Notably, we found that the random selection of genes using differences in expression alone was superior to our proposed selection approach (e.g. fewer genes were identified using random selection) in only 48 out of 10,000 permutations (p = 0.0049), and conclude that the addition of F_ST_ and the published literature search together provides a valuable tool for candidate gene selection.

### RNA Expression Analyses

**Figure 4:**
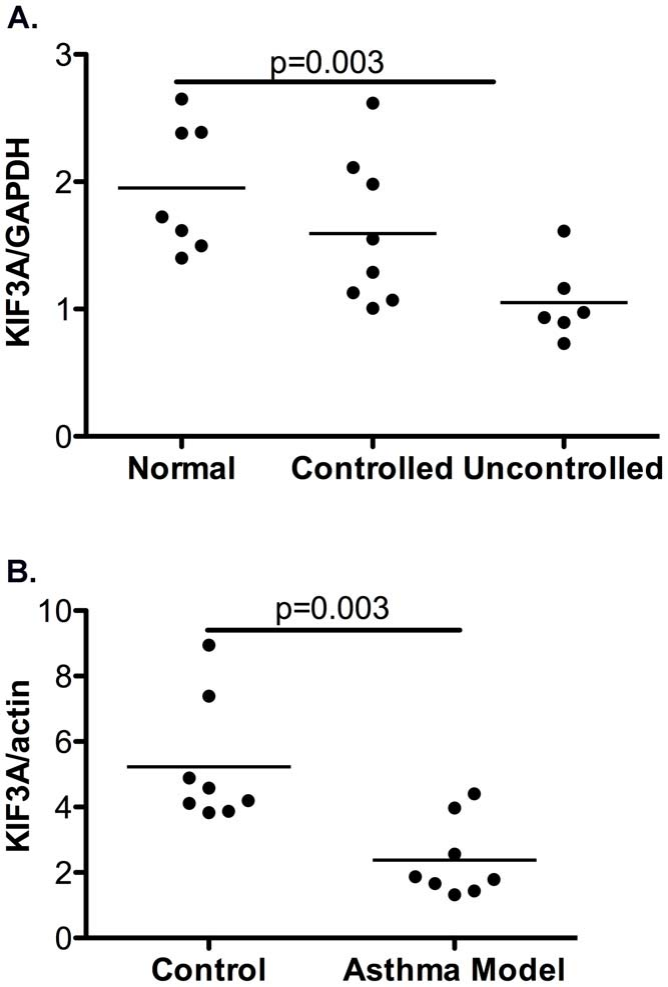
*KIF3A* gene expression of human epithelial RNA from children with uncontrolled asthma and of mouse models with asthma is decreased compared to controls. Gene expression of *KIF3A* in nasal epithelial-derived RNA from non-allergic children, controlled and uncontrolled asthmatics was determined by quantitative PCR. GAPDH was used to normalize for expression. To induce an asthma-like phenotype, wild type Balb/c mice were sensitized and challenged with 100 µg of house dust mite (asthma model) and compared to mice sensitized and challenged with phosphate buffered saline (control). One day post-treatment, RNA was extracted from lungs and gene expression measured by quantitative PCR. Actin was used to normalize for gene expression.

As we found the most significant results with *KIF3A* and as our earlier expression array findings indicate that *KIF3A* expression was down-regulated in nasal epithelial children with acute asthma [6], we wanted to corroborate this observation. To this end, quantitative PCR for *KIF3A* was performed on nasal epithelial RNA samples from the asthmatic children and controls previously included on the array (Fig. 4). Consistent with our array data, we observed that *KIF3A* expression was significantly down-regulated (p<0.05). As an additional independent validation of our results, we also measured expression of *KIF3A* in lung RNA isolated from a house dust mite-induced murine asthma model. Our results indicate that *KIF3A* expression is significantly reduced compared to phosphate buffered saline-treated controls [6] (Fig. 4).

## Discussion

A major challenge for candidate gene association studies is the selection of biologically relevant genes for evaluation. Our unique approach utilized multiple lines of evidence to select relevant candidate genes (Fig. 1). First, as nasal epithelial cells have been previously shown to be a good surrogate for bronchial epithelial cells [10], we took advantage of expression microarray data from these cells to identify genes differentially expressed in asthmatics and non-asthmatic controls. Next, using HapMap, we compared the allelic variation across each of these genes in populations with marked differences in asthma prevalence (CEU vs. CHB), reviewed the published literature to identify specific genes having potential application in the lung and genotyped tagging SNPs across these genes. Using this approach, we determined that SNPs within six genes were associated with asthma (Fig. 2A). Many of these associations were also observed in one or more of our 4 independent populations either at the SNP or gene level (Fig. 2B and Fig. 3), and the association with *KIF3A* was associated in three of four populations examined in addition to our discovery population. The Puerto Rican and Mexican GALA trios not only allowed us to evaluate our results across racial/ethnic groups, but to evaluate genetic associations in populations with potentially different environmental exposures.

For candidate gene selection, researchers have used gene expression results [6], F_ST_ [21], [22], and biologic relevance as gleaned from the literature [1], but no study has combined these strategies. We found that the combination of approaches for candidate gene selection was superior to using one type of data. Indeed, in an independent screen of SNPs in genes found to have dysregulated epithelial gene expression in uncontrolled asthmatics, we found that SNPs with high F_ST_ values were more likely to be associated with asthma than those with low F_ST_ values, including the six genes evaluated in this study, supporting the validity of our gene selection approach. Further, when we randomly selected genes from those that were differentially expressed, we found our selection method was statistically superior. These data point to a strong relationship between epithelial cells and asthma and substantiate the validity of this approach to identify genetic biomarkers of complex disease. Collectively, our approach may be applicable to other complex diseases that show varying prevalences across human populations, and may be a useful tool to select novel gene candidates for assessment of disease associations in other pertinent cell types and tissues.

Using this approach, we have identified *KIF3A* as a possible susceptibility gene for childhood asthma and allergic disease. We found that seven of nine *KIF3A* SNPs genotyped were significantly associated with asthma in our discovery Caucasian population and in independent Caucasian and African American populations – all from Cincinnati (Table 4). SNPs in *KIF3A* were also to a lesser degree, associated with other allergic diseases independent of asthma demonstrating the importance of appropriate phenotyping and selection of controls in genetic studies of asthma (Table 5). If we had merely compared asthmatics to non-asthmatics without determining the non-asthmatics allergic status, we might have missed the association with *KIF3A*. Importantly, all of the disease associated *KIF3A* SNPs and neither of the two non-associated *KIF3A* SNPs displayed significant population differentiation in HapMap CEU and CHB populations (individual F_ST_ values>0.25; Table 4). The observed association was strongest for *KIF3A* rs7737031 which had a PAR for asthma of >18%. Carriers of rs7737031 have more than double the odds of having asthma. Similar to the PAR of this *KIF3A* variant, the PAR for myocardial infarction was 21% in individuals with the previously reported rs10757278 variant located in adjacent *CDKN2A* and *CDKN2B* genes [39]. We also directly genotyped this SNP and evaluated its association with asthma in Puerto Rican and Mexican populations in the GALA Study. Puerto Ricans and Mexicans living in the US have higher and lower asthma prevalences, morbidity, and mortality rates than Caucasians, respectively. Interestingly, our analysis reveals that *KIF3A* rs7737031 was significantly associated in Puerto Ricans and not Mexicans.

**Table 5:** *KIF3A* SNP associations with allergic disease.

*KIF3A*		Allergic vs. Non-allergic Controls^a^			
		Discovery Caucasian		African American	
Frequency cases/controls =		220/246		129/39	
SNP	Major/Minor Allele	OR	P-value	OR	P-value
	In Caucasians				
rs12186803	G/A	1.84	**0.003**	2.11	**0.013**
rs1080001	A/G	1.84	**0.003**	2.11	**0.013**
rs7737031	C/T	1.83	**0.004**	2.11	**0.013**
rs9784675	A/G^b^	1.72	**0.008**	2.13	**0.009**
rs3798130	G/A^b^	1.83	**0.003**	1.89	**0.023**
rs2299011	C/G^b^	1.82	**0.003**	1.92	**0.017**
rs12514685	C/T^b^	1.82	**0.004**	1.85	**0.023**
rs1468216	G/A	0.70	0.076	0.78	0.416
rs17691077	A/C	0.91	0.661		

a The discovery Caucasian population and African American population consisted of asthmatic children from the GCPCR and non-asthmatic/non-allergic controls from the GCPCR and GCC. Associations between allergic children and controls were tested using an additive model. Odds ratios (OR) were determined using logistic regression based on the minor allele after adjusting for age, gender and population stratification. Bolding indicates the SNP associations <0.05.

b Indicates major and minor alleles are reversed in African Americans.

KIF3A is a heterotrimeric member of the kinesin superfamily of microtubule associated motors that are important in the transport of protein complexes within cilia and flagella [40], [41] among other roles. Cilia, together with mucus and the airway surface liquid layer, make up the mucociliary apparatus that clears inhaled allergens or other particles from the lung. Defective mucociliary clearance is a characteristic feature of several genetically linked airway diseases including asthma and cystic fibrosis [42], [43], yet the mechanisms responsible for poor mucus and/or allergen clearance from the airways remain largely unknown.

As *KIF3A* is located on 5q31, immediately upstream of *IL-4*, we also noted a strong LD between *IL-4* and *KIF3A* SNPs in our discovery cohort, a finding that is consistent with another published report [44]. Examination of International HapMap data [45] indicates that this LD exists across many other populations as well making it difficult to determine if one or both genes confer risk. Numerous studies have reported associations with *IL-4*, asthma and other allergic diseases [29], [46]. It is possible that some of the previous asthma associations reported between asthma and *IL-4* may reflect the LD with *KIF3A*. Therefore, we further examined the biologic plausibility of *KIF3A* as an asthma susceptibility gene by examining gene expression in the lungs of mice. We found expression of *KIF3A* was significantly reduced in HDM-treated mice compared to controls (Fig. 4). We speculate that in asthmatics specifically, diminished *KIF3A* expression might be important in allowing the lung to repair itself after exacerbation. Alternatively, diminished *KIF3A* expression may contribute to the lungs' inability to clear mucus and remove inhaled particles and aeroallergens therefore exacerbating the asthma and/or allergic phenotypes. Individuals with polymorphic cilia genes may have further reduced cilia gene expression, diminished ciliary function and increased allergen exposure resulting in even greater susceptibility to asthma and allergic disease. Regardless of the mechanism of action, *KIF3A* is clearly down-regulated in asthma, supporting a role for this novel gene in the pathogenesis of this disease. While our analyses of tagging SNPs provide strong evidence of a gene-disease association, it will be important to investigate the combined effects of *IL-4* and *KIF3A* in future studies and to identify specific causal variants in *KIF3A*.These findings emphasize the importance of evaluating multiple genes and critically exploring the biological relevance of genes previously unknown to influence disease susceptibility.

In summary, our study took advantage of our previously published evaluation of nasal epithelial cell-derived RNA from asthmatic and non-allergic children [6], population differences in asthma prevalence, tagging SNPs in the HapMap database, and the published literature to identify six genes (*ADCY2*, *DNAH5*, *KIF3A*, *PDE4B*, *PLAU*, *SPRR2B*) for detailed and targeted genetic testing. The most strongly associated gene, *KIF3A*, was first reported by our group as having a role in asthma in 2009 [47]. KIF3A has also been associated with aspirin sensitive asthma [48]. Here, we verify that *KIF3A* is a novel candidate gene for childhood asthma and show that its expression is down regulated in nasal epithelial cells in asthmatics. Our success supports the validity of our approach for identifying asthma candidate genes with a high likelihood of exhibiting association in specific variants. As the level of genomic data continues to increase, it will be imperative to develop methods which can help focus studies. By demonstrating that we can identify associations using this approach and that these associations can be replicated, we have provided a novel framework for the identification of candidate genes.
